# Description of an early Cretaceous termite (Isoptera: Kalotermitidae) and its associated intestinal protozoa, with comments on their co-evolution

**DOI:** 10.1186/1756-3305-2-12

**Published:** 2009-02-18

**Authors:** George O Poinar

**Affiliations:** 1 Department of Zoology, Oregon State University, Corvallis, Oregon, 97331, USA

## Abstract

**Background:**

The remarkable mutualistic associations between termites and protists are in large part responsible for the evolutionary success of these eusocial insects. It is unknown when this symbiosis was first established, but the present study shows that fossil termite protists existed in the Mesozoic.

**Results:**

A new species of termite (*Kalotermes burmensis *n. sp.) in Early Cretaceous Burmese amber had part of its abdomen damaged, thus exposing trophic stages and cysts of diverse protists. Some protists were still attached to the gut intima while others were in the amber matrix adjacent to the damaged portion. Ten new fossil flagellate species in the Trichomonada, Hypermastigida and Oxymonadea are described in nine new genera assigned to 6 extant families. Systematic placement and names of the fossil flagellates are based on morphological similarities with extant genera associated with lower termites. The following new flagellate taxa are established: *Foainites icelus *n. gen. n. sp., *Spiromastigites acanthodes *n. gen. n. sp., *Trichonymphites henis *n. gen., n. sp., *Teranymphites rhabdotis *n. gen. n. sp., *Oxymonas protus *n. sp., *Oxymonites gerus *n. gen., n. sp., *Microrhopalodites polynucleatis *n. gen., n. sp., *Sauromonites katatonis *n. gen., n. sp., *Dinenymphites spiris *n. gen., n. sp., *Pyrsonymphites cordylinis *n. gen., n. sp. A new genus of fossil amoeba is also described as *Endamoebites proterus *n. gen., n. sp. Fourteen additional trophic and encystid protist stages are figured and briefly characterized.

**Conclusion:**

This represents the earliest fossil record of mutualism between microorganisms and animals and the first descriptions of protists from a fossil termite. Discovering the same orders, families and possibly genera of protists that occur today in Early Cretaceous kalotermitids shows considerable behaviour and morphological stability of both host and protists. The possible significance of protist cysts associated with the fossil termite is discussed in regards the possibility that coprophagy, as well as proctodeal trophallaxis, was a method by which some termite protozoa were transferred intrastadially and intergenerationally at this time.

## Background

Termites are one of the most successful eusocial insect groups today and certainly the most notorious as a result of their damage to human dwellings. Their success can be attributed in large part to microbial (especially protozoa and bacterial) symbionts harbored in their alimentary tract. Especially important are gut protists, which are essential for the survival of termites feeding on lignocelluloses. While termites do produce endogenous cellulases from salivary glands and gut cells, cellulolytic protists are crucial for the complete digestion of cellulose in wood-feeding termites [1-3]. In the lower termites, these symbionts are mostly flagellates belonging to the Oxymonadida, Trichomonada and Hypermastigida [4-7]. Flagellates associated with an Early Cretaceous lower termite of the family Kalotermitidae are described and compared with mutualistic flagellates of extant kalotermitids. This discovery shows that, while the protist species represent different genera and species, mutualistic associations between protists and termites had already been established some 100 million years ago. The present study represents the earliest fossil record of mutualism between microorganisms and animals [8].

## Results

### Description of host termite

Damage to the termite included the loss of the left hind wing, the tip of the abdomen (including the cerci), the right side of the metathorax and the right side of the first three abdominal segments. All wings were flexed at their bases. However, it was possible to determine the venation in the distal three quarters of the right forewing and the complete right hind wing.

Based on the morphological characters of the termite (Sc absent from the hind wing, R simple in both wings, no reticulation between the veins posterior to R, R separate from the costal margin, presence of a number of regularly spaced oblique forward branches between the Rs and C), the fossil is placed in the family Kalotermitidae [9,10].

Its small size, low number of antennal segments, strongly sclerotized radial sector, unsclerotized M and Cu veins, complete M vein positioned halfway between Rs and Cu, wing membrane densely covered with minute, pigmented nodules, tibial spurs lacking on the shaft of the mid-tibia and the presence of ocelli and arolia, align the fossil with the genus *Kalotermes *Hagen 1853 [9,10]. Since the fossil differs from previously described termites in Burmese amber [10-13], it is described below as a new species. It should be noted that the placement of the fossil in the extant genus *Kalotermes *is tentative since some diagnostic characters (number of apical spines on the tibiae, front wing venation, etc.) were obscured.

Family Kalotermitidae Banks 1919

Genus *Kalotermes *Hagen 1853

**Figure 1:**
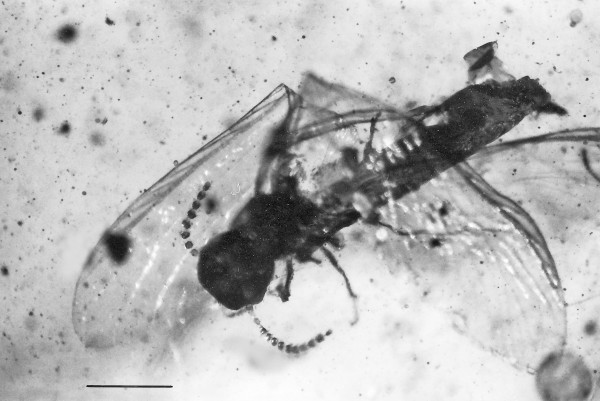
**Dorsal view of *Kalotermes burmensis *n. sp**. Bar = 690 μm.

**Figure 2:**
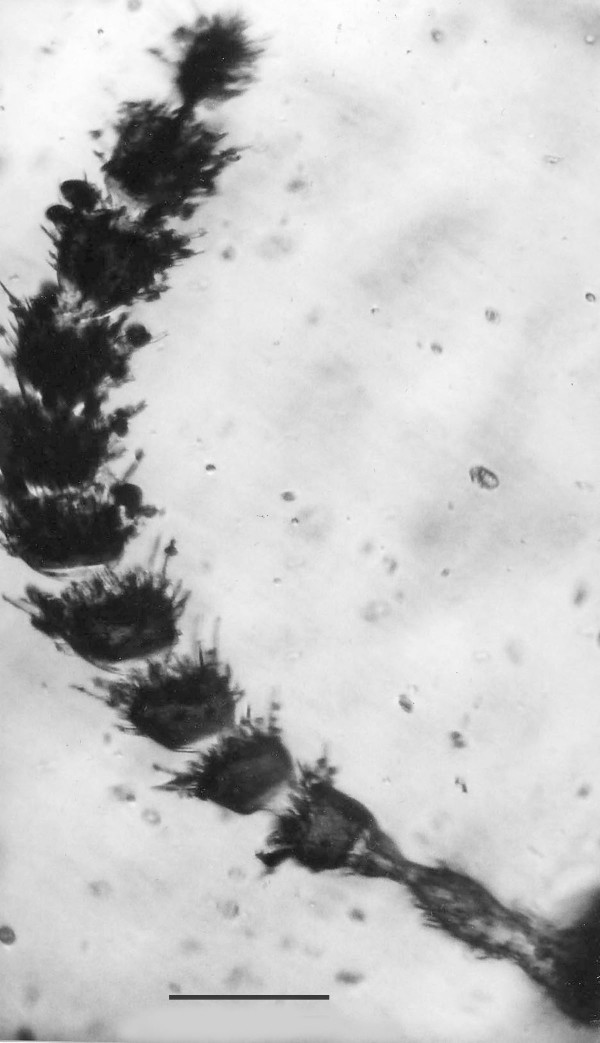
**Left antenna of *Kalotermes burmensis *n. sp**. First antennomere partially hidden by head. Bar = 106 μm.

**Figure 3:**
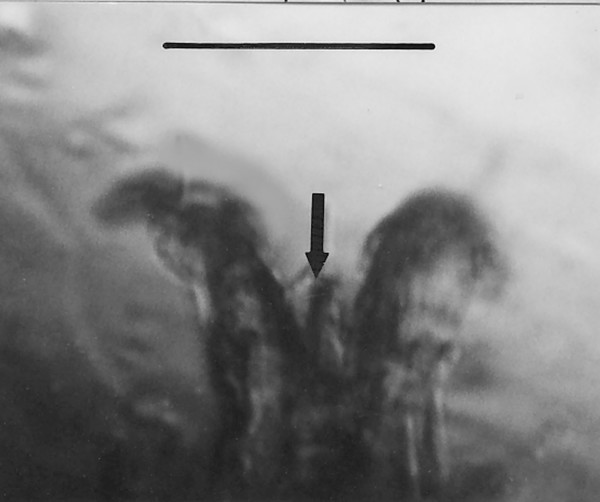
**Claw with arolium (arrow) of *Kalotermes burmensis *n. sp**. Bar = 63 μm.

**Figure 4:**
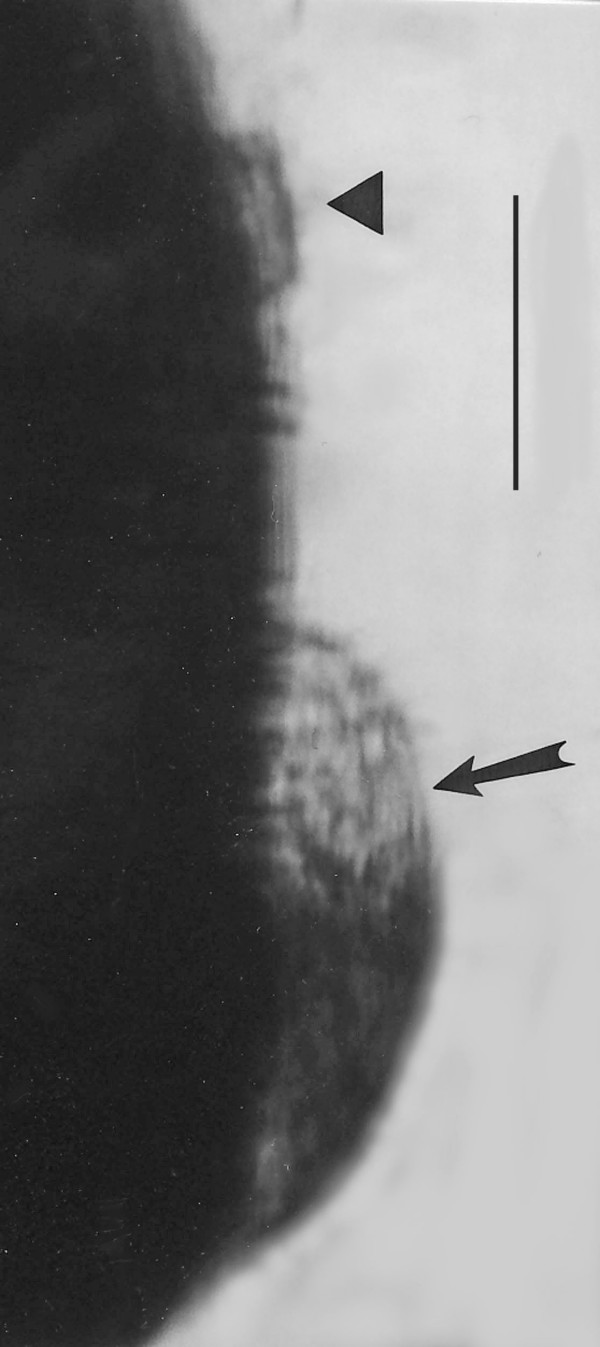
**Lateral view of head of *Kalotermes burmensis *n. sp**. showing ocellus (arrowhead) and compound eye (arrow). Bar = 50 μm.

**Figure 5:**
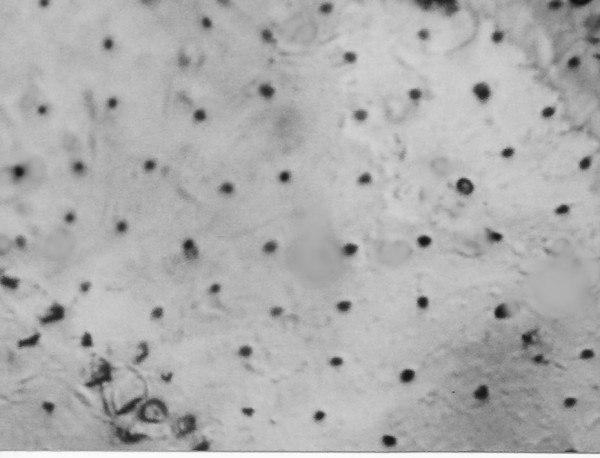
**Minute black nodules covering wing membrane of *Kalotermes burmensis *n. sp**.

**Figure 6:**
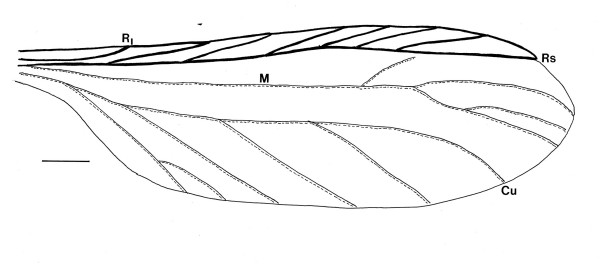
**Hind wing of *Kalotermes burmensis *n. sp**. showing radius (R_1_), radial sector (Rs), medial (M), and cubital (Cu) veins. Bar = 328 μm.

*Kalotermes burmensis *n. sp. (Figs. 1, 2, 3, 4, 5 and 6)

Alate (sex unknown): Fontanelle absent; head capsule, thorax, legs and abdomen dark brown; length of body (from anterior edge of clypeus to end of abdomen) 3.3 mm; head length, 567 μm; head width, 607 μm; eye diameter, 113 μm; ocellus small, 30 μm in diameter; located 62 μm from compound eye, length terminal maxillary palp, 135 μm; length terminal labial palp, 108 μm; right mandible with prominent terminal tooth and smaller subterminal tooth; left antennae 850 μm in length, with 12 segments, terminal segment smallest; maximum length of pronotum, 525 μm; minimum length of pronotum, 420 μm; width of pronotum, 672 μm; length scale on fore wing, 531 μm; length scale on hind wing, 292 μm; length right hind wing, 3.9 mm; width right hind wing, 1.2 mm; venation on right hind wing as follows: Sc not present; R simple, short; M with double fork near terminus; wing membrane covered with minute black nodules; 6 branches extend from Rs to C; all branches of cubitus simple except basal one; apical spines on tibia partially obstructed, at least 2 on meso and metatibia; tarsi four-jointed with first three segments short; claws 95 μm in length; arolium short; cerci missing.

Holotype: Specimen B-I-2 deposited in the Poinar amber collection maintained at Oregon State University.

Locality: Amber mine in the Hukawng Valley, southwest of Maingkhwan in the state of Kachin (26°20'N, 96°36'E) in Myanmar (Burma).

Comments: The present species differs from *Kalotermes swinhoei *[11] and *K. tristis *[12], previously described from Burmese amber. While Emerson [14] and Krishna [9] felt that *K. swinhoei *and *K. tristis *could be synonymous, Williams [10] considered both as valid species and presented diagnostic characters to separate them. The absence of arolia, simple M vein, wing membrane lacking nodules and number of antennal segments separates *K. tristis *from the present species. From *K. swinhoei*, *K. burmensis *differs in its slightly smaller size (3.3 mm vs 3.8 mm), greater eye diameter (250 μm vs 113 μm), larger head length (607 μm vs 530 μm), shorter pronotal width (672 μm vs 760 μm), shorter terminal maxillary palp (135 μm vs 190 μm), shorter terminal labial palp (108 μm vs 160 μm), fewer number of antennal segments (12 vs at least 16), shorter hind wing (3.9 mm vs 4.8 mm), length of R vein in hind wing (about 1/5 of length in *K. burmensis *and 1/3 of length in *K. swinhoei *and approximately equal width of the head and pronotum in *K. burmensis*, while the width of the pronotum (760 μm) is larger than the head (530 μm) in *K. swinhoei*.

The wing venation, head shape, presence and size of arolia, presence of ocelli, absence of spines on the shaft of the mid-tibia, number of tarsal and antennal segments and presence of pigmented wing nodes separates *K. burmensis *from the four Burmese amber specimens described by Engel et al, [13], as well as all of the Tertiary species of kalotermitids [14].

### Description of protists

A variety of protists were associated with *K. burmensis*. Some were still attached to the gut lining, while others were free in the amber matrix adjacent to the exposed gut. Those that revealed morphological characters similar to protist families and genera found in extant lower termites are described below. Since the amber matrix normally contains a variety of small, oval-spherical bodies, care was taken to select for descriptions only protozoa attached to the gut intima or with features aligning them with extant groups associated with lower termites. None of the forms presented below are considered to be insect parasites or pathogens [15]. Stages that could not be identified due to insufficient characters are documented with photographs and briefly characterized.

Except for one species described in the extant genus *Oxymonas*, all of the protists are placed in fossil genera, with names based on extant genera with similar morphological characters. However, it is acknowledged that since not all diagnostic characters could be determined in the fossil protists and features that were present could align the fossil with more than one extant protist lineage, the similarities so indicated between the fossils and extant genera are only tentative.

Terminology and classification generally follows that of Brugerolle and Lee [4,5] and Patterson et al. [16]. A synopsis of the described fossils with their higher level systematic positions are summarized below.

Phylum Parabasalia

   Class Trichomonada Kirby

      Order Trichomonadida Kirby

         Family Devescovinidae Doflein

**Figure 7:**
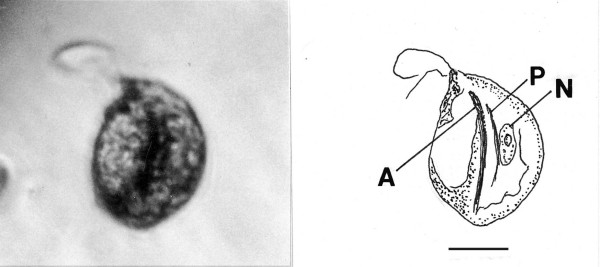
**Photo and drawing of *Foainites icelus *n. gen. n. sp**. N = nucleus, A = axostyle, P = parabasal. Bar = 12 μm.

            *Foainites icelus *n. gen., n. sp. (Figs. 7A,B)

   Class Hypermastigida Grassi & Foá

      Order Spirotrichonymphida Light

         Family Holomastigotidae Grassi

**Figure 8:**
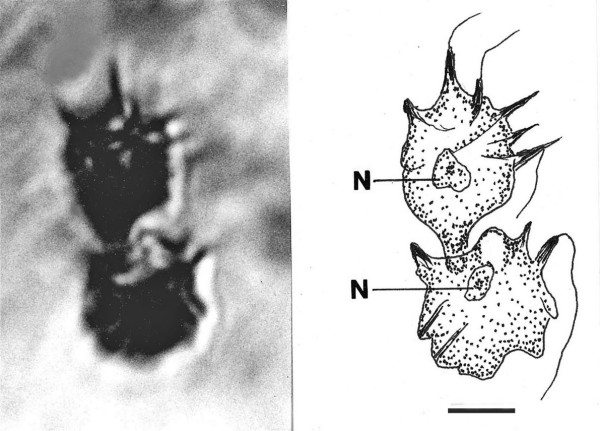
**Photo and drawing of *Spiromastigites acanthodes *n. gen., n. sp**. N = nucleus. Bar = 5 μm.

            *Spiromastigites acanthodes *n. gen., n. sp. (Figs. 8A,B)

      Order Trichonymphida Poche

         Family Trichonymphidae Kent

**Figure 9:**
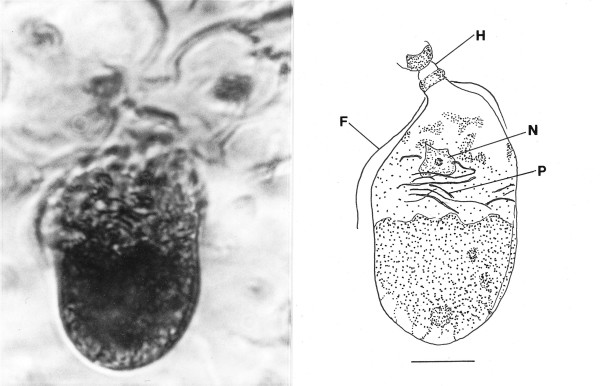
**Photo and drawing of *Trichonymphites henis *n. gen., n. sp**. H = holdfast, F = rostral flagellum, n = putative nucleus. Bar = 11 μm.

            *Trichonymphites henis *n. gen., n. sp. (Figs. 9A,B)

         Family Teranymphidae Koidzumi

**Figure 10:**
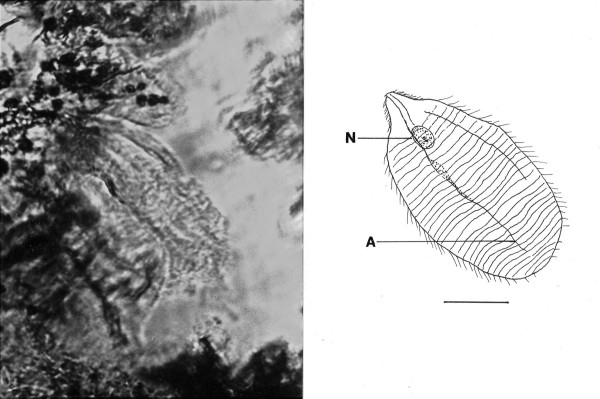
**Photo and drawing of *Teranymphites rhabdotis *n. gen. n. sp**. Left portion of body covered by a second specimen. N = nucleus, A = putative axostyle, P = parabasal. Bar = 24 μm.

            *Teranymphites rhabdotis *n. gen., n. sp. (Figs. 10A,B)

Phylum Oxymonada

   Class Oxymonadea Grassi

      Order Oxymonadida Grassi

         Family Oxymonadidae Kirby

            *Oxymonas *Janicki

**Figure 11:**
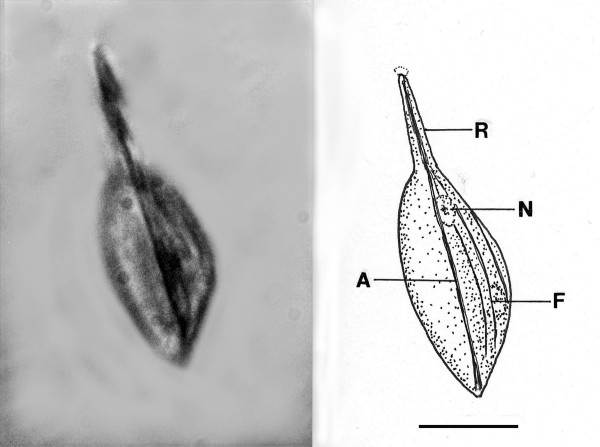
**Photo and drawing of *Oxymonas protus *n. sp**. R = rostellum, N = nucleus, A = putative axostyle, F = fibrous structure. Bar = 15 μm.

               *O. protus *n. sp. (Figs. 11A,B)

**Figure 12:**
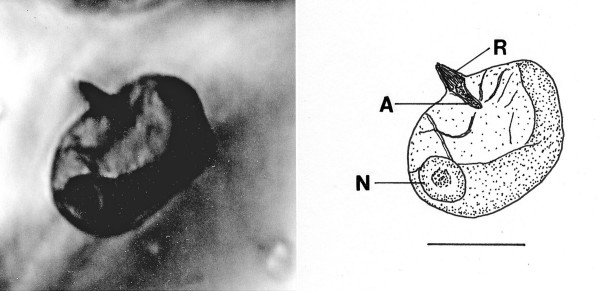
**Photo and drawing of *Oxymonites gerus *n. gen., n. sp**. R = rostellum, A = axostyle, N = nucleus. Bar = 26 μm.

            *Oxymonites gerus *n. gen., n. sp. (Figs. 12A,B)

**Figure 13:**
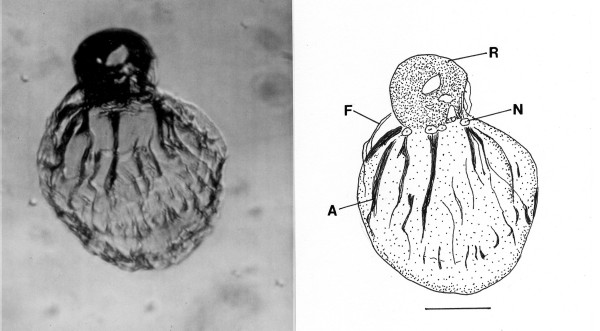
**Photo and drawing of *Microrhopalodites polynucleatis *n. gen., n. sp**. R = rostellum, A = axostyle, N = nucleus, Bar = 41 μm.

            *Microrhopalodites polynucleatis *n. gen., n. sp.(Figs. 13A,B)

**Figure 14:**
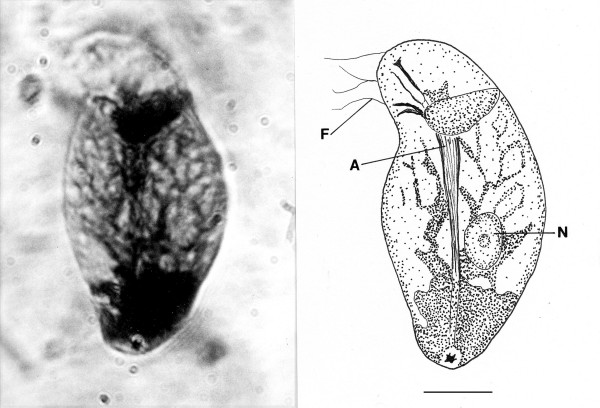
**Photo and drawing of *Sauromonites katatonis *n. gen., n. sp**. F = flagellum, A = axostyle, N = nucleus. Bar = 22 μm.

            *Sauromonites katatonis *n. gen., n. sp. (Figs. 14A,B)

         Family Pyrsonymphidae Grassi

**Figure 15:**
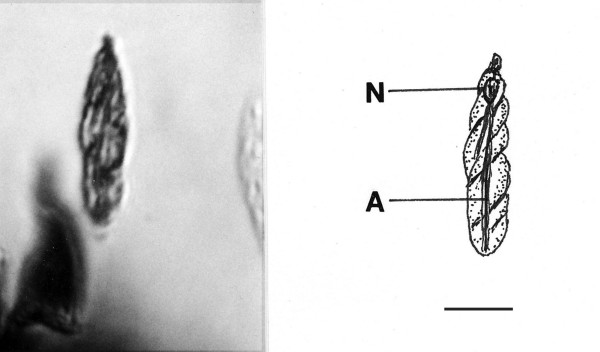
**Photo and drawing of *Dinenymphites spiris *n. gen., n. sp**. N = nucleus, A = axostyle, Bar = 13 μm.

            *Dinenymphites spiris *n. gen., n. sp. (Figs. 15A,B)

**Figure 16:**
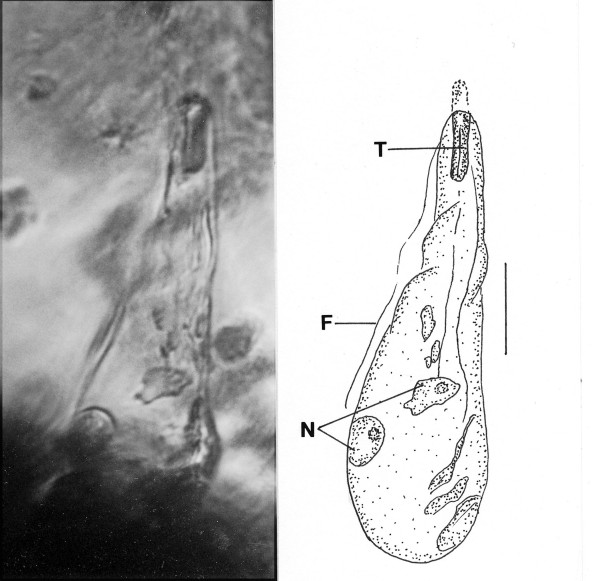
**Photo and drawing of *Pyrsonymphites cordylinis *n. gen., n. sp**. F = flagellum, T = tubular area, N = nucleus. Bar = 21 μm.

            *Pyrsonymphites cordylinis *n. gen., n. sp. (Figs. 16A,B)

"Sarcodina"

Amoeba of uncertain affinity

**Figure 17:**
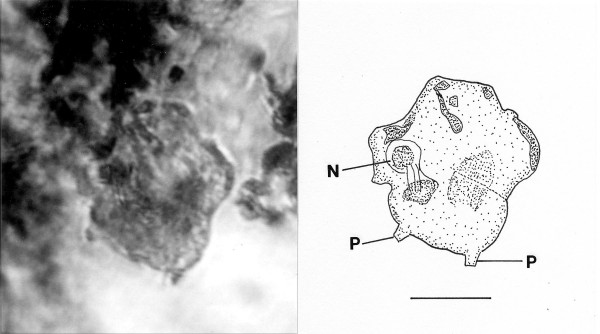
**Photo and drawing of *Endamoebites proterus *n. gen., n. sp**. P = protuberance, N = nucleus dividing. Bar = 14 μm.

            *Endamoebites proterus *n. gen., n. sp. (Figs. 17A,B)

Family Devescovinidae Doflein, 1911

*Foainites *Poinar, n. gen. (Figs. 7A,B)

Description. Body asymmetrical; two anterior flagella (more could be present) arising from anterior area; trailing flagellum apparently in front of body; nucleus located in middle of body; parabasal body straight, not coiled around nucleus; axostyle extending through body.

Type species. *Foainites icelus *n. sp.

*Foainites icelus *Poinar, n. sp.

Description. Length, 31 μm; width, 23 μm; length of longest flagellum, 24 μm; parabasal body rod-shaped; axostyle not protruding from body.

Etymology: From the Greek "ikelos" meaning similar, in regards to the body shape resembling extant species of *Foaina *Janicki.

Holotype: Specimen (accession # B-I-2) deposited in the Poinar amber collection maintained at Oregon State University.

Locality: Amber mine in the Hukawng Valley, southwest of Maingkhwan in Kachin state (26°20'N, 96°36'E), Myanmar (Burma).

Comments: The fossil resembles the body shape of extant species of *Foaina *Janicki 1915, however, it could be related to one of the other genera of small devescovinids. The straight parabasal body slightly longer than the nucleus distinguishes it from extant species. All known species of *Foaina*, whose size range extends from 6 μm to 59 μm, occur in kalotermitids [5,9].

Family Holomastigotidae Grassi, 1892

*Spiromastigites *Poinar n. gen. (Figs. 8A,B)

Description. Body bearing emergent processes (spines), some of which bear flagella at their tips; axostyle not observed; rostrum absent; nucleus positioned at midbody.

Type species: *S. acanthodes *n. sp.

*Spiromastigites acanthodes *Poinar n. sp.

Description. Two adjacent individuals, upper individual (17 μm in length) with terminal process connected to girdle of lower individual (14 μm in length).

Holotype: Specimen B-I-2 deposited in the Poinar amber collection maintained at Oregon State University.

Locality: Amber mine in the Hukawng Valley, southwest of Maingkhwan in the state of Kachin (26°20'N, 96°36'E) in Myanmar (Burma).

Etymology: From the Greek "akantha" for thorn, in reference to the processes on the body surface.

Comment: These two fossils are difficult to classify. Their spiraled flagellar rows and absence of an axostyle and rostrum show similarities to members of the extant genus *Spiromastigotes *Duboscq & Grassé, 1943, but the nucleus is anterior in the latter genus. It is not known whether the pair are conjugating or in the final stage of binary fission. The type species of *Spiromastigotes *is 10–20 μm in length and occurs in the hodotermitid, *Anacanthotermes ochraceus *(Brugerolle & Lee, 2000b).

Family Trichonymphidae Kent, 1880

*Trichonymphites *Poinar n. gen. (Figs. 9A,B).

Description. Body short and broad; rostrum short; holdfast attached to intima of termite intestine; rostral flagella (only 2 visible) extend slightly over one half body length; axostyle not seen; putative nucleus positioned in upper third of body; parabasal bodies ribbon-shaped, haphazardly positioned in area surrounding nucleus.

Type species: *T. henis *n. sp.

*T. henis *Poinar n. sp.

Description. Body acorn-shaped, length, 49 μm; width, 25 μm; posterior portion of body apparently filled with endoplasmic inclusions.

Holotype: Specimen B-I-2 deposited in the Poinar amber collection maintained at Oregon State University.

Locality: Amber mine in the Hukawng Valley, southwest of Maingkhwan in the state of Kachin (26°20'N, 96°36'E) in Myanmar (Burma).

Etymology: From the Greek "henos" for old.

Comment: This genus is placed in the family Trichonymphidae based on its relatively short rostral flagella, absence of an axostyle, and ribbon-like parabasals associated with the nucleus. One of three cysts of this or a related species in the same family is shown in Fig. 19. Members of the genus *Trichonympha *occur in at least 16 termite genera worldwide, including *Kalotermes *[9,17,18]. Because of its wide distribution in the termite families Hodotermitidae, Rhinotermitidae and Kalotermitidae, Kirby [17] suspected that species of *Trichonympha *were already present in the various termite lineages when they first appeared and then underwent a period of co-evolution that is continuing today.

**Figure 18:**
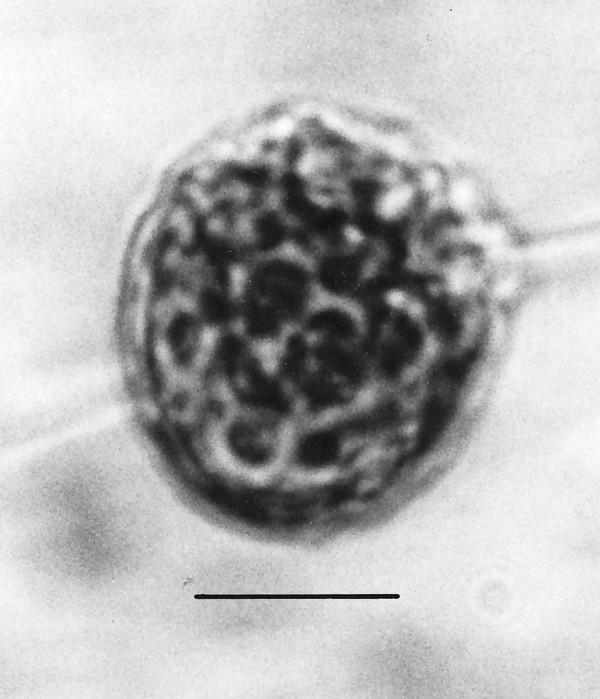
**Possible amoebic cyst.** Bar = 24 μm.

Family Teranymphidae Koidzumi, 1917

*Teranymphites *Poinar n. gen. (Figs. 10A,B)

Description. Medium sized oval cell, with short rostrum attached to intestinal cells of host; rostral flagella short; post-rostral area with parallel flagellar rings without connections and giving the cell a segmented appearance; nucleus located under rostral area; putative axostyles (possibly 2) narrow, fiber-like.

Type species: *T. rhabdotis *n. sp.

*Teranymphites. rhabdotis *Poinar, n. sp.

Description: Body length, 84 μm; body width, 46 μm; rostrum with rows of short flagella; post-rostral area with at least 30 parallel rows of flagella separated by ectoplasmic bands; parabasal body short.

Holotype: Specimen B-I-2 deposited in the Poinar amber collection maintained at Oregon State University.

Locality: Amber mine in the Hukawng Valley, southwest of Maingkhwan in the state of Kachin (26°20'N, 96°36'E) in Myanmar (Burma).

Etymology: From the Greek "rhabdotos" for lined.

Comment: Flagella inserted on parallel rows separated by ectoplasmic bands is a condition found in members of the extant genus *Teranympha *Koidzumi 1917. The long rostral flagella that also characterize this genus may be hidden since the anterior end of the fossil is attached to the gut intima. Long, narrow, fibril axostyles occur in the related extant genus *Spirotrichosoma *Sutherland 1933 and both genera have an anterior nucleus. Extant species of *Teranympha *and *Spirotrichosoma *range from 90–270 μm and 55–395 μm in length, respectively, and occur in members of the families Rhinotermitidae and Termopsidae, respectively [5].

Family Oxymonadidae Kirby, 1928

*Oxymonas *Janicki, 1915

*Oxymonas protus *n. sp. (Figs. 11A,B)

Description. Body spindle shaped; length (including rostellum), 50 μm, width, 15 μm, length rostellum,17 μm; anterior nucleus approx 7 μm in longest dimension, positioned at base of rostellum; body with supporting fibers attached at base of rostellum; single axostyle extends length of body; faint holdfast at tip of rostellum.

Holotype: Specimen B-I-2 deposited in the Poinar amber collection maintained at Oregon State University.

Locality: Amber mine in the Hukawng Valley, southwest of Maingkhwan in the state of Kachin (26°20'N, 96°36'E) in Myanmar (Burma).

Etymology: From the Greek" protos" for first regarding its fossil status.

Comment: The presence of an anterior rostellum with associated fibers and the uninucleate condition are diagnostic characters of the genus *Oxymonas*. However members of *Oxymonas *usually have 4 flagella arising from the shoulder area, which are not evident in the fossil [4,19]. Species of *Oxymonas *occur in at least 10 genera of kalotermitids and range from 5–165 μm in length [5,9]. The holdfast is used to attach the cell to the chitinous lining of the termite gut and the size of the rostellum has been used to estimate the density of populations in termite guts, with a lengthy rostellum indicating a crowded condition [19].

*Oxymonites *Poinar n. gen. (Figs.12A,B)

Description. Uni-nucleated flagellate with body comprising a single karyomastigont; body wider than long; axostyle short, not extending more than half body length; nucleus large, located near middle of body; rostellum with several fiber bundles.

Type species: *O. gerus *n. sp.

*Oxymonites gerus *Poinar n. sp.

Description. Length, 41 μm; width, 51 μm; rostellum short, 8 μm in length; rostellum without associated fibrous structures; nucleus spherical, 11 μm in diameter.

Holotype: Specimen B-I-2 deposited in the Poinar amber collection maintained at Oregon State University.

Locality: Amber mine in the Hukawng Valley, southwest of Maingkhwan in the state of Kachin (26°20'N, 96°36'E) in Myanmar (Burma).

Etymology: From the Greek "geros" for old age.

**Figure 19:**
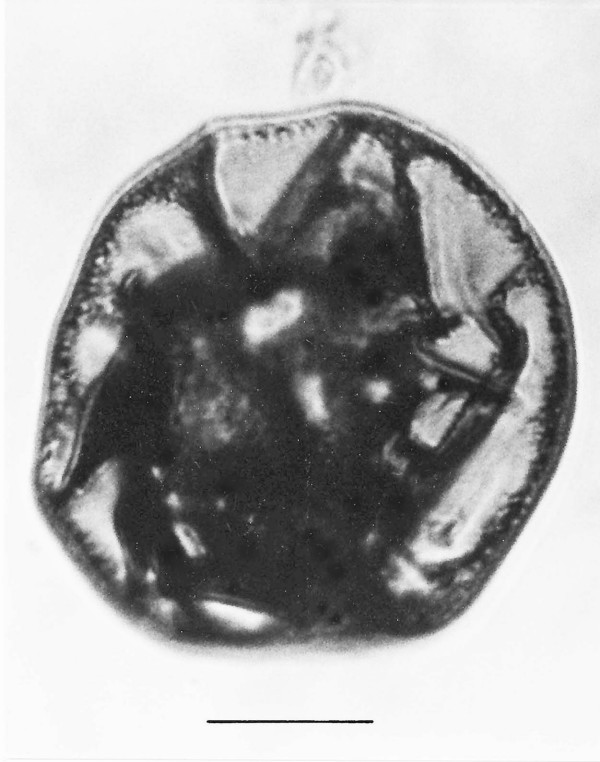
**Large spherical cyst with two nuclei.** Bar = 28 μm.

**Figure 20:**
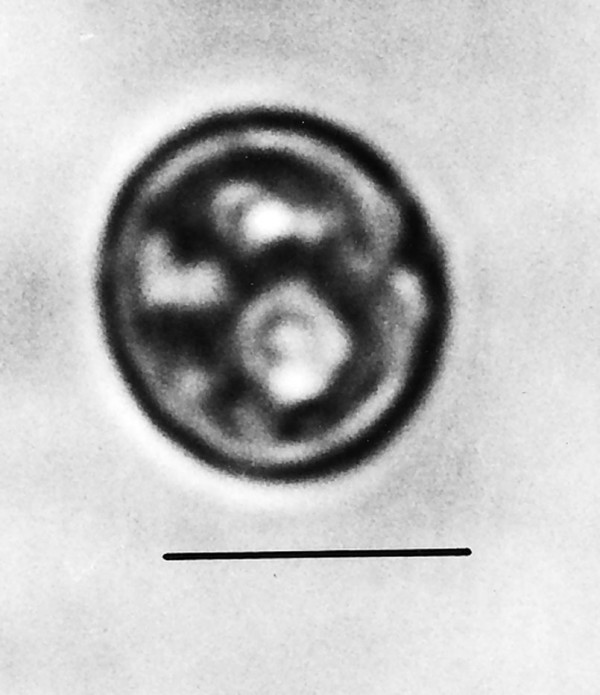
**Small spherical cyst with a pair of nucleated cells**. Bar = 14 μm.

Comments: The presence of an anterior rostellum with fibers arising from the base of the attachment point and the single nucleus is why this fossil was placed in the Oxymonadidae. While oxymonidids typically have an anteriorly placed nucleus, during certain phases, the nucleus may be located in the posterior portion of the body [19]. The shape and size of the rostellum vary greatly in extant oxymonadids [19]. The family is widely distributed in kalotermitids, with some 27 species described worldwide [9]. Possible cysts of this fossil and/or *Oxymonas protus *are shown in Figs. 19 and 20. Each cyst contains a nucleus and the one in Fig. 19 shows an axostyle in the lower portion of the body, which is characteristic of some oxymonadid cysts [20].

*Microrhopalodites *Poinar n. gen. (Figs. 13A,B)

Description. Large multinucleated flagellate with corona containing several karyomastigotes with small, anteriorly positioned nuclei; base of rostral area with filaments that extend posteriorly as axostyles; flagella attached at base of rostellum extend at least to midbody; rostral area hemi-spherical in shape.

Type species: *M. polynucleatis *n. sp.

*M. polynucleatis *Poinar, n. sp.

Description: Large cell, with knob-shaped anterior rostral area; total length, 159 μm; greatest width, 116 μm; length rostral area, 47 μm; width rostral area, 52 μm; at least 3 small nuclei positioned at base of rostral area; flagella attached to corona area beneath base of rostellum.

Holotype: Specimen B-I-2 deposited in the Poinar amber collection maintained at Oregon State University.

Locality: Amber mine in the Hukawng Valley, southwest of Maingkhwan in the state of Kachin (26°20'N, 96°36'E) in Myanmar (Burma).

Etymology: From the Greek "poly" indicating many nuclei.

Comment: The corona of several karyomastigotes with small nuclei, the large size and the modified rostral area align the fossil with the extant genus *Microrhopalodina *Grassi & Foa. This genus contains four extant species ranging in size from 20 μm -165 μm and all occur in kalotermitid termites [4]. It is difficult to say if the rounded rostral region is characteristic of the mature trophozoite or if the cell was entering a resting stage. Superficially, this fossil has the appearance of an eugregarine sporont, with the rostral area corresponding to the protomerite and the basal portion the deutomerite. However, there is no scar on the top of the hemispherical rostral area that would indicate the attachment of an epimerite, no evidence of a septum separating the two parts and no evidence of a large nucleus in the portion that would correspond to the deutomerite. The putative flagella and small nuclei associated with the rostral area are not characteristic of eugregarine sporonts, certainly not those from extant termites [21,22].

*Sauromonites *Poinar n. gen. (Figs. 14A,B).

Description. Axostyle prominent, thickened anteriorly, extending length of body; 4 flagella emerging from rostral area; nucleus located in lower half of body; large dark body at base of rostral area (macronucleus?); network of apparent fiber bundles throughout body; minute dark area at posterior end may represent tip of axostyle.

Type species: *Sauromonites katatonis *sp. n.

*Sauromonites katatonis *Poinar sp. n.

Description. Length,106 μm; width approximately 1/2 length; length rostral area, 27 μm; width at base of rostral area, 36 μm; nucleus large, greatest diameter, 13 μm.

Holotype: Specimen (accession # B-I-2) deposited in the Poinar amber collection maintained at Oregon State University.

Locality: Amber mine in the Hukawng Valley, southwest of Maingkhwan in the state of Kachin (26°20'N, 96°36'E) in Myanmar (Burma).

Etymology. From the Greek "katatonos" meaning broader than high.

Comments: This species is roughly the same size and shape as extant species of *Sauromonas *in kalotermitids [4].

Family Pyrsonymphidae Grassi, 1892

*Dinenymphites *Poinar n. gen. (Figs. 15A,B)

Description. Small rod-shaped, spindle cell with at least 4 spirally twisted flagellar cords adhering to body; small holdfast; axostyle slender; nucleus in anterior portion of body.

Type species: *Dinenymphites spiris *n. sp.

*Dinenymphites spiris *Poinar, n. sp.

Description. Body slender, 41 μm in length, 9 μm in width; axostyle extending length of cell.

Holotype: Specimen B-I-2 deposited in the Poinar amber collection maintained at Oregon State University.

Locality: Amber mine in the Hukawng Valley, southwest of Maingkhwan in the state of Kachin (26°20'N, 96°36'E) in Myanmar (Burma).

Etymology: From the Greek "speira" for twisted in reference to the body structure.

Comments: The fossil resembles extant species of the genus *Dinenympha *Leidy, which range from 24 μm to 64 μm in length. Members of this genus are now considered motile forms of *Pyrsonympha *Leidy [4].

*Pyrsonymphites *Poinar, n. gen. (Figs. 16A,B)

Description. Large club-shaped, slightly spirally twisted flagellate with two nuclei positioned slightly below mid-body; with several flagella (some adhering to cell body) arising from anterior end of cell; anterior tip with thickened tubular area; axostyle not detected.

Type species: *P. cordylinis *n. sp.

*Pyrsonymphites cordylinis *Poinar, n. sp.

Description. Length, 152 μm; width, 48 μm; length longest flagellum, 98 μm; length cephalic tube, 24 μm; tubular area (holdfast?) inserted in host's intestinal intima.

Holotype: Specimen B-I-2 deposited in the Poinar amber collection maintained at Oregon State University.

Locality: Amber mine in the Hukawng Valley, southwest of Maingkhwan in the state of Kachin (26°20'N, 96°36'E) in Myanmar (Burma).

Etymology: From the Greek " kordylinas" for club-shaped in reference to the shape of the fossil.

Comment: This fossil resembles the extant *Pyrsonympha *Leidy regarding the position of the flagella and posterior nucleus. The two cells in the lower body appear to be nuclei. Attached pyriform cells of *Pyrsonympha *range between 150 μm and 200 μm, which is within the size of the fossil. *Dinenymphites spiris *could be the unattached stage of this or another species of *Pyrsonympha*. Members of *Pyrsonympha *occur today in members of the Rhinotermitidae [4].

"Sarcodina"

Amoebae of uncertain affinities (Patterson et al., 2000).

*Endamoebites *Poinar n. gen. (Figs. 17A,B).

Description. Spherical nucleus in process of dividing; nucleolus not apparent; body amoeboid-like, nearly spherical, with broad pseudopodia and short protuberances.

Type species: *Endamoebites proterus *n. sp.

*Endamoebites proterus *Poinar n. sp.

Description. Greatest diameter, 33 μm; nucleus undergoing division; diameter of upper nucleus, 8 μm; endoplasm containing particles of various sizes.

Holotype: Specimen B-I-2 deposited in the Poinar amber collection maintained at Oregon State University.

Locality: Amber mine in the Hukawng Valley, southwest of Maingkhwan in the state of Kachin (26°20'N, 96°36'E) in Myanmar (Burma).

Etymology: From the Greek "proteros" for earlier.

**Figure 21:**
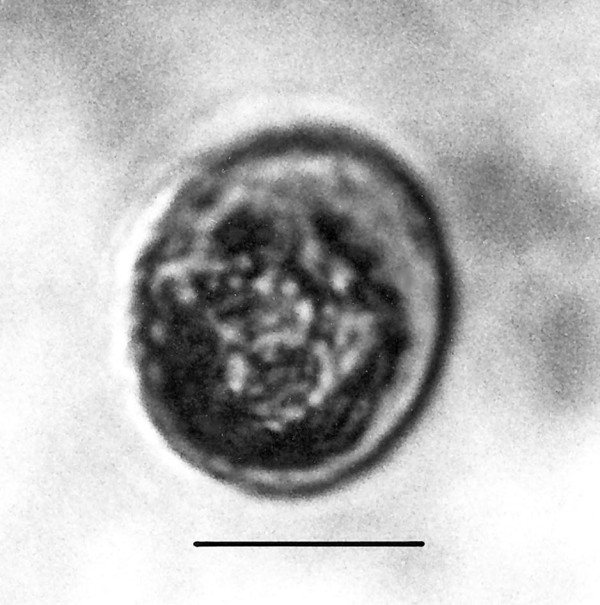
**Spherical cyst with two or more nuclei**. Bar = 14 μm.

Comments: The size and shape of the body and nucleus resemble those of extant species of *Endamoeba *Leidy from termites and roaches [23] which are the only known hosts [16]. The nucleus appears to be dividing and some putative chromatin threads connect the two adjacent nuclear zones. A possible cyst of *Endamoebites *(Fig. 21) is 53 μm in diameter, possesses a thick membrane, contains 13 nuclei in the focal plane shown and resembles cysts of extant *Endamoeba *[16].

### Additional protists

**Figure 22:**
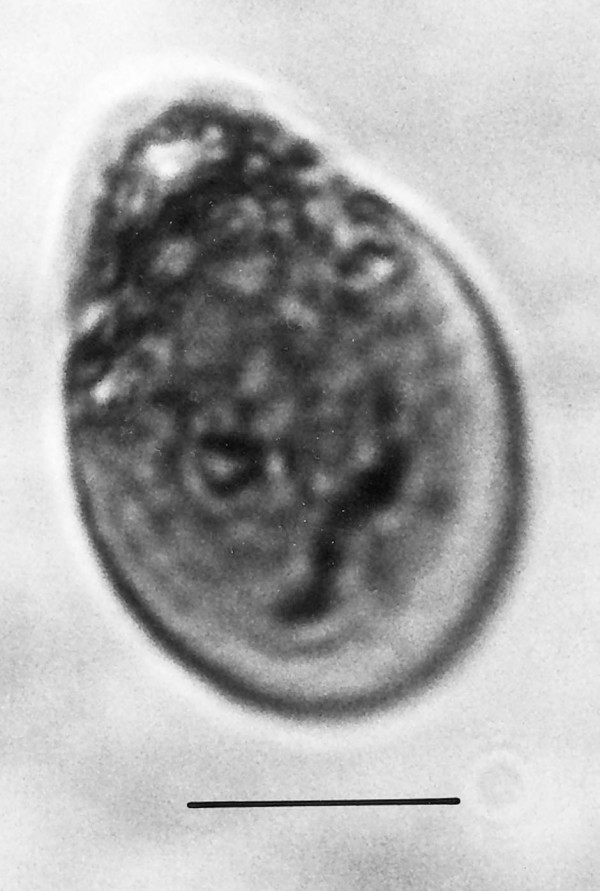
**Pyriform cyst**. Bar = 12 μm.

**Figure 23:**
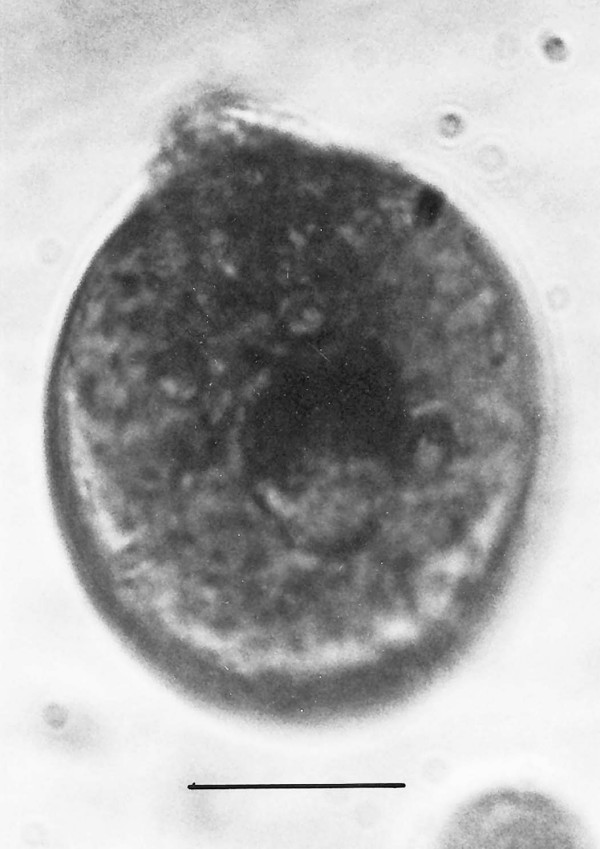
**Spherical cyst**. Bar = 14 μm.

**Figure 24:**
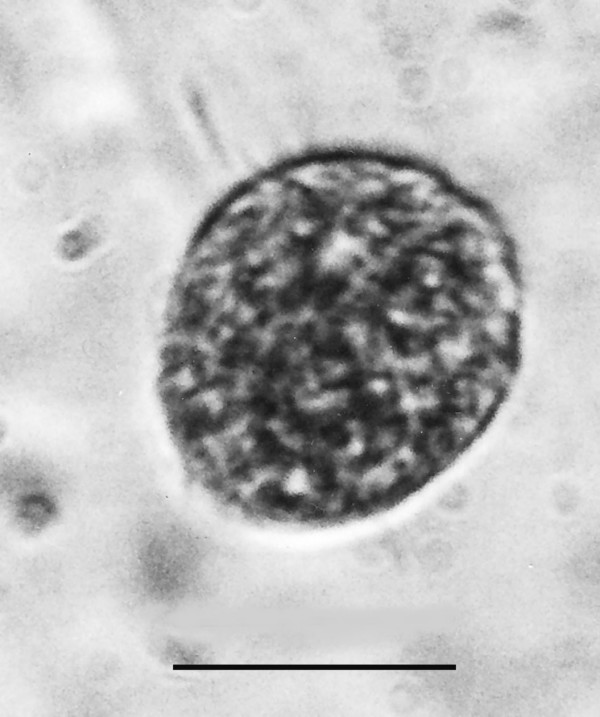
**Possible ciliate**. Bar = 14 μm.

**Figure 25:**
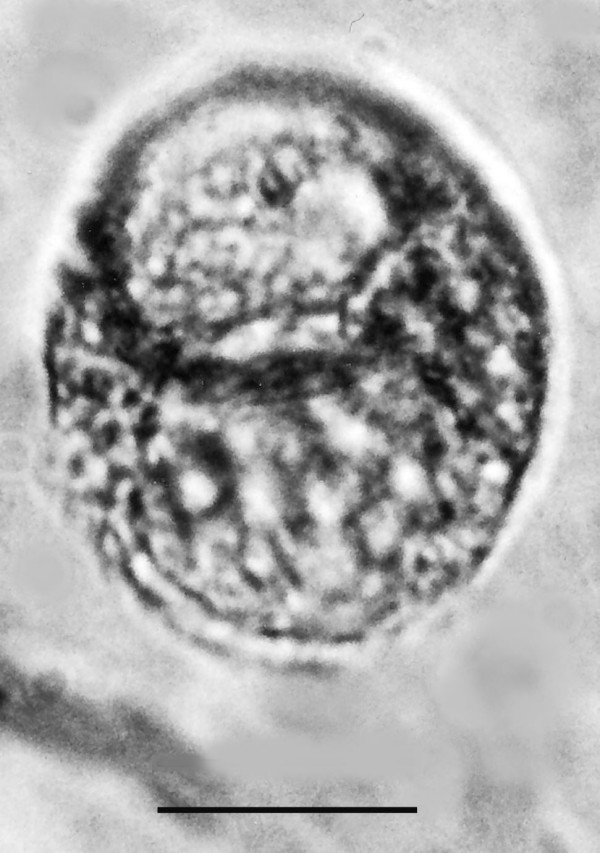
**Possible ciliate**. Bar = 12 μm.

**Figure 26:**
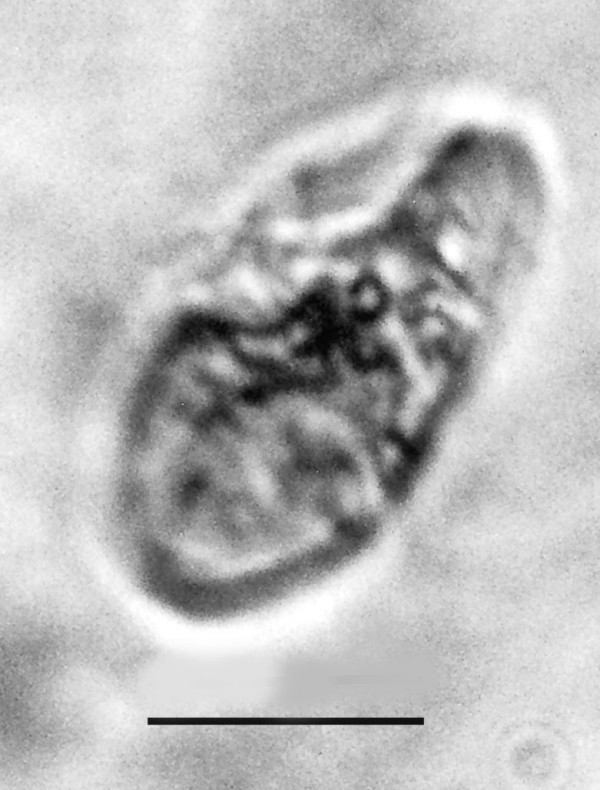
**Possible ciliate**. Bar = 14 μm.

**Figure 27:**
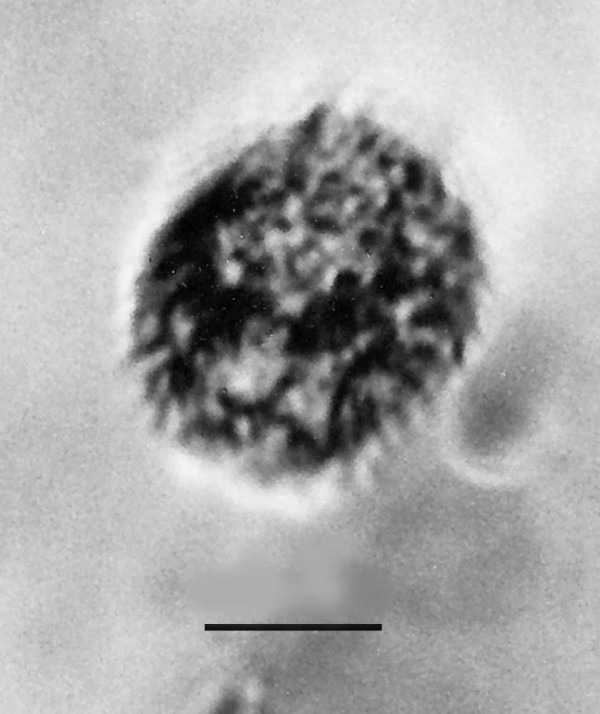
**Possible ciliate**. Bar = 10 μm.

**Figure 28:**
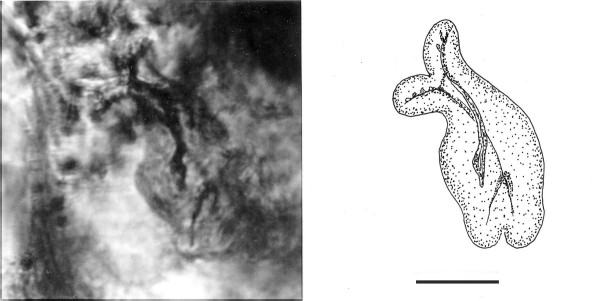
**A, B. Photo and drawing of a dividing cell with a double rostral area, each with an axostyle**. Specimen partially embedded in gut of host. Bar = 12 μm.

**Figure 29:**
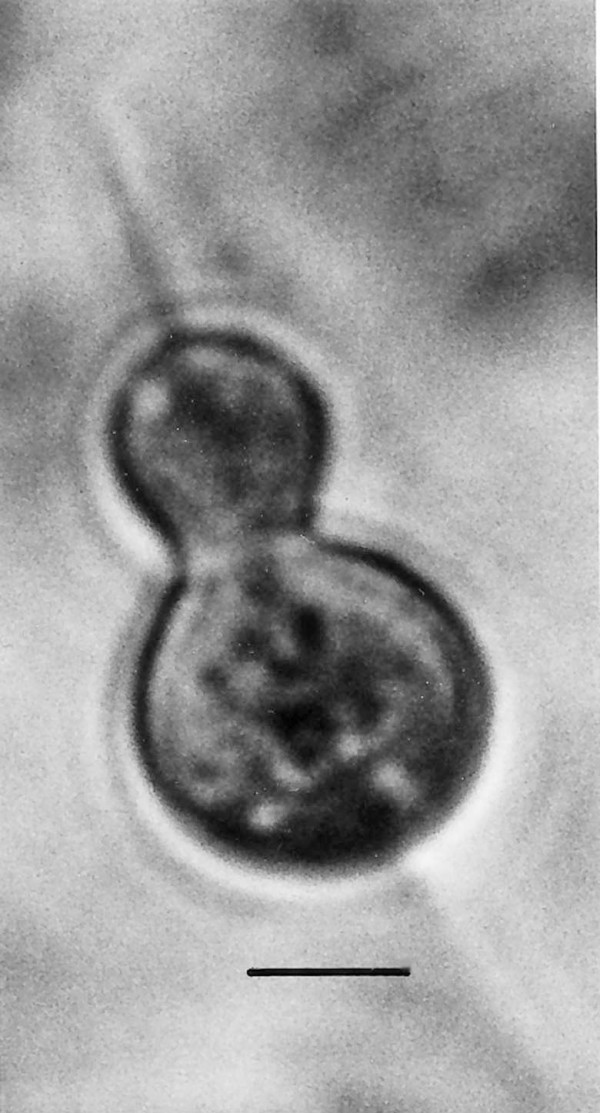
**Example of double cell with one mastigont being extruded**. Bar = 6 μm.

**Figure 30:**
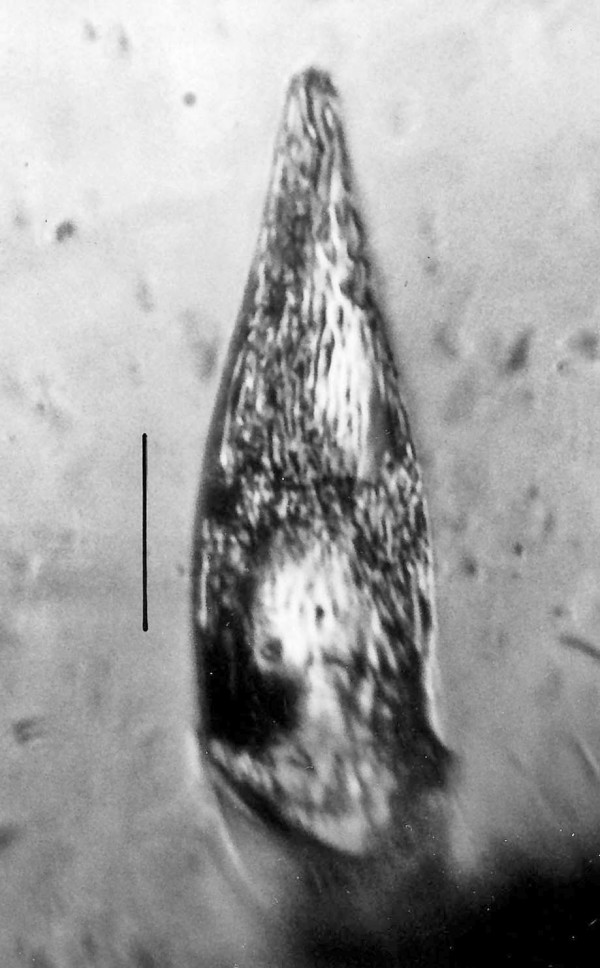
**Unidentified flagellate, probably of the family Trichonymphidae**. Bar = 28 μm.

**Figure 31:**
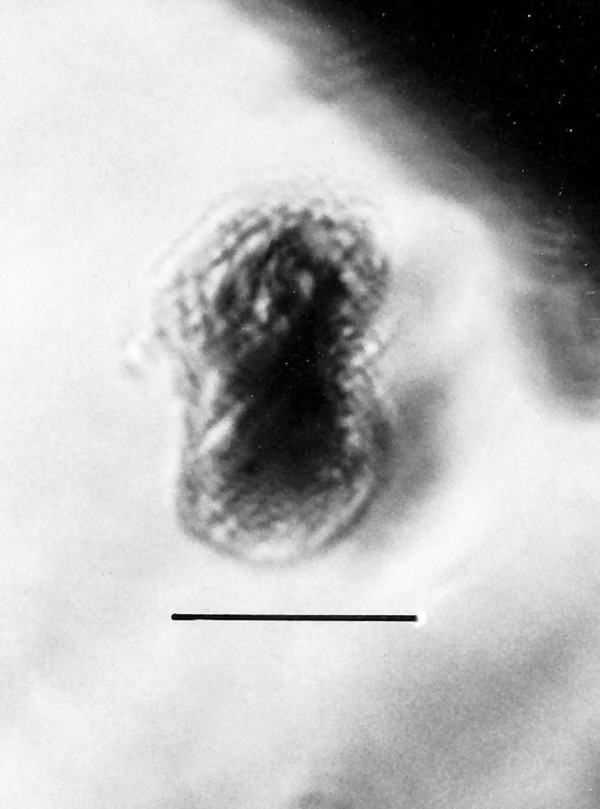
**Cell undergoing binary fission**. Bar = 12 μm.

**Figure 32:**
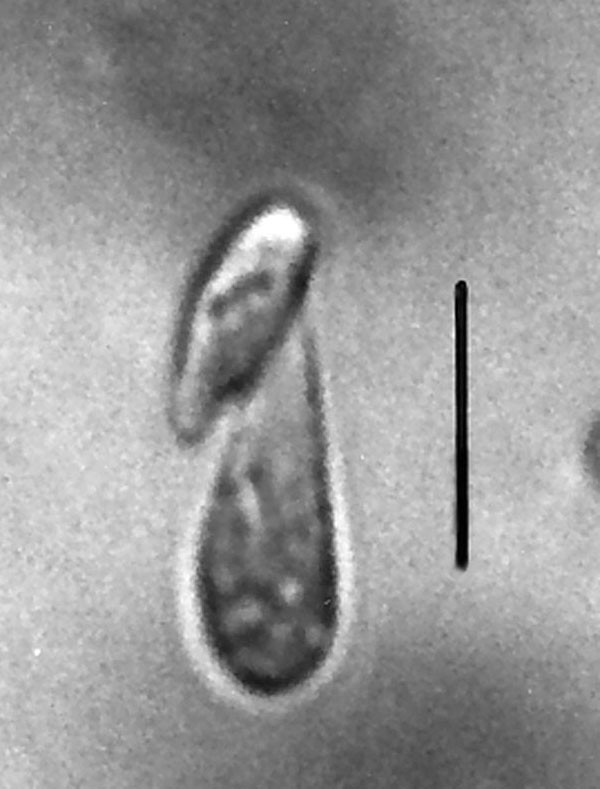
**A pair of elliptical cells**. Bar = 13 μm.

Additional unknown protist stages associated with *K. burmensis *are illustrated in Figs. 18, 19,20, 22, 23, 24, 25, 26, 27, 28, 29, 30, 31 and 32. Encysted forms are shown in Figs. 18, 19, 20, 21, 22 and 23. The large (97 μm in diameter), bi-nucleated, walled cyst in Fig. 19 greatly resembles the binucleated cysts of *Trichonympha *described from *Cryptocercus *cockroaches by Cleveland et al. [[6], pg. 205, Fig. 13b]. Since these cysts were found in the amber matrix adjoining the termite, it is likely that they were already formed when the termite was entombed, especially since Cleveland et al. [6] showed that they required a period of 4 days to form in *Cryptocercus*. It is highly unlikely that any of the cysts were formed after the termite entered the resin, since terpinols and other chemicals would have killed the pre-cystic, trophic stages instantly.

Unknown stages, some of which could be ciliates, are shown in Figs. 24, 25, 26, 27, 28 and 29, a flagellate in Fig. 30, a pair of small elliptical cells in Fig. 29 and protists undergoing division in Figs. 30, 31 and 32. Ciliates have been reported from extant termites [24] but have not been well documented.

A pair of dauer juvenile rhabditid nematodes were also associated with *K. burmensis*. These will be described in a separate study.

## Discussion

The present study represents the first descriptions of protists from a fossil termite and the earliest fossil record of mutualism involving microorganisms and animals [8]. The occurrence of an Early Cretaceous kalotermitid termite with a variety of flagellates that includes members of the same orders, families and possibly genera that occur today in kalotermitids shows considerable behavior and morphological stability of both host and protists.

Successful establishment of protozoa in xylophagous insects necessated particular attributes. The protozoa had to withstand the chemical and physical conditions inside the insect's alimentary tract, utilize the gut contents as a food source, cause no damage to the host, and to be carried through successive insect stages and generations. These protists apparently underwent a long period of co-evolution with their hosts, since some lineages found today in the intestines of xylophagous cockroaches and lower termites are thought to have been established in Carboniferous Blattida [25].

Today, the composition of intestinal protozoa tends to be correlated with the phylogenetic position of their termite hosts and it is possible to classify families, genera and even species of termites based on their flagellates [7]. This also applies to *K. burmensis*, since it contains representatives of the three most abundant and widespread groups of protozoa in kalotermitids today (Trichomonada, Hypermastigida and Oxymonadea). If the systematic assignments are correct, some of the protists in *K. burmensis *appear to be restricted to other families of lower termites today. However in the Early Cretaceous, partitioning of host habitats was probably less fixed and the choice of host by a protozoan was predicated largely on availability and chance encounter. Some of the protozoa described here may have been thwarted by a change in the habits of the host and since they could not adapt, disappeared from the colonies. Since angiosperms were becoming more diverse by the mid-Cretaceous, some protists in *K. burmensis *may have succumbed when the host diet shifted from gymnosperm to angiosperm wood. While it is assumed that most of the protozoa described here had a mutualistic relationship with *K. burmensis*, as members of the Trichomonada, Hypermastigida and Oxymonadida do with extant lower termites, some, as for instance *Endamoebites proterus*, may have been simply commensals and provided no benefit to *K. burmensis*.

One striking difference in the behavior of the protist symbionts in extant termites and *K. burmensis *is the presence of encysted stages in the latter. In extant termites, mature protist cysts are rarely formed [26,27] and never in alates since the flagellates are transferred to nest mates and hatchlings by proctodeal trophallaxis (intrastadial and intergenerational transfer). However it has been hypothesized that the distant ancestor of termites passed flagellate cysts to nest mates and hatchlings by coprophagy [6,7,27-30].

Several possible scenarios could explain the presence of cysts in *K. burmensis*:

A), the colony was subsocial and ingesting cysts in fecal pellets (coprophagy) was the main method for the intrastadial and intergenerational transfer of protists; B), the colony was subsocial or eusocial and both coprophagy (ingesting cysts in fecal pellets) and proctodeal trophallaxis served to transfer the flagellates to nest mates and hatchlings; C), the colony was eusocial and protozoa were transferred by proctodeal trophallaxis. The cysts were an ancestral carry-over and represent an evolutionary dead-end; D), some of the cysts could have been ingested while feeding and had no trophic association with *K. burmensis*.

## Materials and methods

### Specimens

The amber with the fossil termite containing the protists is roughly semi-circular in outline, measuring 13 mm along the longest edge, 10 mm in width and 1 mm in thickness. Observations, drawings and photographs were made with a Nikon SMZ-10 R stereoscopic microscope and Nikon Optiphot compound microscope. Since all photographs were taken through the thickness of the amber matrix, it was not possible to get as close as desired to individual protists without polishing away adjacent ones as well as portions of the termite host. Therefore all photos were taken at 20×. With such a small image, it was only possible to obtain a single clear photo of the specimen since further fine adjustment produced blurry images. Adobe Photoshop was used to enlarge the photos and to obtain several modified images by using different settings of contrast, light intensity and resolution. The drawings were made from a combination of the modified images and that is why they contain more detail than the corresponding photographs, which represent the best single image obtained under the various settings. Thus Photoshop manipulation was used to replace optical sections, that were not possible to make with such small objects and at such a low magnification.

### Locality

The amber was obtained from a mine first excavated in 2001 in the Hukawng Valley, southwest of Maingkhwan in the state of Kachin (26°20'N, 96°36'E) in Myanmar (Burma). On the basis of paleontological evidence, this new Noije Bum 2001 Summit Amber Site was assigned to the Early Cretaceous, Upper Albian [31] placing the age at 97 to 110 mya.

### Type material

In accordance with section 8.6 of the ICZN's International Code of Zoological Nomenclature, copies of this article are deposited at the following five publicly accessible libraries: Natural History Museum, London, UK; American Museum of Natural History, New York, USA; Museum National d'Histoire Naturelle, Paris, France; Russian Academy of Sciences, Moscow, Russia; Academia Sinica, Taipei, Taiwan.

### Source

Nuclear magnetic resonance (NMR) spectra and the presence of araucaroid wood fibers in amber samples from the Noije Bum 2001 Summit site indicate an araucarian (possibly *Agathis*) tree source for the amber [32].

## Competing interests

The author declares that he has no competing interests.
